# Evaluation of Tunable Data Compression in Energy-Aware Wireless Sensor Networks

**DOI:** 10.3390/s100403195

**Published:** 2010-04-01

**Authors:** Beihua Ying, Yongpan Liu, Huazhong Yang, Hui Wang

**Affiliations:** Department of Electronic Engineering, Tsinghua National Laboratory for Information Science and Technology, Tsinghua University, Beijing 100084, China; E-Mails: ypliu@tsinghua.edu.cn (Y.L.); wangh@tsinghua.edu.cn (H.W.); yingbh04@mails.tsinghua.edu.cn (B.Y.)

**Keywords:** wireless sensor networks, data compression, evaluation index, energy efficiency

## Abstract

Energy is an important consideration in wireless sensor networks. In the current compression evaluations, traditional indices are still used, while energy efficiency is probably neglected. Moreover, various evaluation biases significantly affect the final results. All these factors lead to a subjective evaluation. In this paper, a new criterion is proposed and a series of tunable compression algorithms are reevaluated. The results show that the new criterion makes the evaluation more objective. Additionally it indicates the situations when compression is unnecessary. A new adaptive compression arbitration system is proposed based on the evaluation results, which improves the performance of compression algorithms.

## 1. Introduction

Wireless sensor networks (WSNs), a new network structure, have received continuous attention in recent ten years. Since the 1990s, when sensor networks emerged as a fundamentally new tool for military monitoring, nowadays they are widely used in many application fields such as agriculture, ecosystems, medical care and smart homes, especially for regions which are inaccessible or unattended. By right of the essential function in data collection, WSNs connect the physical environment with human beings [1].

Generally, each sensor node transmits monitoring data over its corresponding path to the sink. Since the nodes are battery-operated and no fixed infrastructure exists, energy becomes the primary concern in such networks. Moreover, the number of nodes in WSNs can be extremely large. It is prohibitively difficult to replace or recharge them to extend the operational lifetime of network. Thus, energy efficiency is considered as the major metric which impacts network performance significantly. Many advances have been made with the purpose of enhancing network lifetime [2,3].

Among different applications, continuous data collection for environmental monitoring is relatively popular [4]. In this scenario, sensor nodes continuously sample surrounding physical phenomena and return them to the sink. The ubiquity of redundancies in the data inspires researchers to introduce compression technology for reducing data volume and saving communication energy costs. Recent developments propose many challenges for data processing and the related technologies.

Lots of compression methods are designed specifically for sensor networks. However, it seems to be difficult to get proper advice about which one is more suitable for a certain application. The lack of research on data compression evaluation and the corresponding criteria make it hard to provide efficient guidelines for both algorithm design and application. Besides, various kinds of evaluation bias tend to lead to inaccurate conclusions, which then leads to wrong choices.

In this paper, we study current compression algorithms for WSNs, and propose a novel evaluation criterion which is more applicable for them. The main contributions of our work are threefold:

First, a new evaluation criterion is presented to give attention to the energy efficiency of compression implemented in the sensor nodes. Since energy consumption is one of the most important design metrics in WSNs, this criterion will do well in such compression evaluation to provide useful suggestions during both design and application.

Second, current tunable compression algorithms aimed at WSNs are reevaluated in depth at the node level and the network level. Various kinds of real datasets are adopted, which cover almost all types of environmental data. Evaluation results based on our criterion and several traditional indices are compared avoiding different evaluation bias.

Third, based on the results, a novel compression arbitration system is proposed to enhance the performance of compression algorithms by avoiding unnecessary energy losses. Furthermore, several design considerations of compression are discussed. We suggest that design concept of compression algorithms should be changed due to the particularity of WSNs.

The remainder of the paper is structured as follows. Section 2 discusses the related work on both compression algorithms and evaluation methods. Several aspects that impact evaluation results are analyzed. Section 3 presents the principle of evaluation and defines the new criterion. Experiment setup and the methodology are described in Section 4 with the results and corresponding discussions given in Section 5. A new compression arbitration system is presented in Section 6 and Section 7 offers a summary to conclude the paper.

## 2. Related Work

Data compression is regarded as a traditional technology used in digital communication, broadcasting, storage, and multimedia systems. Being applied to WSNs, compression faces more new challenges. Although there have been a number of algorithms proposed for WSNs up to the present [5–25], problems exist in them at the same time. One is how can we select a proper algorithm for a given application among several methods on hand. Reasons that lead to this confusion are discussed.

### 2.1. Limitation in Evaluation Index

Traditional compression is used with the purpose of improving the performances in communication time, transmission bandwidth and storage space. Various evaluation indices are defined. Some of them are extended for use in WSNs:

(1) Compression ratio

Compression ratio is one of the most important design indices in data compression. It visually describes the compression effect of algorithm, and is formulated as a ratio between the volume of the compressed data and the raw one. Based on it, the improvements in communication time, transmission bandwidth and storage space can be quantitatively measured.

In WSNs, compression ratio is also considered as one of the major evaluation criteria. Since it can indicate the reduction of communication energy costs, researchers prefer to show the exciting results produced by their new algorithms [8,9,14,16,17,19,21–24]. Likewise, users would like to choose the algorithm with better compression results in view of lower energy consumptions in data communication.

(2) Compression error

Compression error is another important criterion with various expression forms, such as RMS (Root Mean Square) error, peak error, SNR (Signal to Noise Ratio), and so on. It describes the degree of information loss after compressing.

In WSNs, lossy compression is much more popular due to the better compression ratio. Thus, compression error is unavoidable [14,17,23]. Based on this index, users can assess which compression will get less data distortion or whether it meets the application requirements.

(3) Compression complexity

Compression complexity includes space complexity and time complexity, which represent the costs of hardware resources and execution time in data compressing, respectively. Lower space complexity means less memory occupation required; lower time complexity incurs shorter delays.

Nevertheless, compression complexity has not been seriously considered in WSNs. It is accepted that algorithms with high complexity are unsuitable for sensor nodes with restricted capabilities. Therefore, complexity seems more like a qualitative criterion. Users pay more attention to the feasibility of the algorithm, rather than the real costs of storage and time. Only in some specific applications, has compression complexity been quantitatively investigated [7,22].

In a word, researchers still prefer to use traditional standards for data compression evaluation in WSNs. Compression ratio seems the main criterion for choosing a more satisfied algorithm. However, as mentioned above, saving energy is the fundamental purpose in sensor networks. Each criterion listed above only partially reflects energy information. Thus, a new criterion is urgently desired for WSNs, though the existing ones are doing well in traditional compression evaluation.

### 2.2. Existence of Evaluation Bias

During compression evaluating, several kinds of bias will directly influence the results. Among them, data bias and execution bias are two main aspects.

Data bias appears when non-uniform experimental datasets are used for the comparison of algorithms. It is well known that datasets with different characteristics will produce greatly different test results. For instance, data with higher redundancy trend to a lower compression ratio. So, it is difficult to distinguish which one improves the observed compression performance: the test data or the algorithm itself.

Unfortunately, data bias is ubiquitous in compression evaluation. Designers use their own datasets [5,7,10–13,19–21,24,25], most of which are unpublished. This causes confusion during algorithm selection. Hence, removing data bias in evaluation is important. We consider using uniform and open datasets as the most direct and simple way. On the other hand, execution bias has a similar impact on evaluation. It will probably happen that different time overhead is taken even if the same algorithm is realized. Many factors affect the result such as coding style, test platform, compilation tool, and so on [7,9–13,16,18–23,25]. Compared to data bias, the execution one is difficult to avoid. For all that, it is also wished to remove it as much as possible.

We list a series of compression algorithms and their related information in Table 1. All of them are specifically designed for WSNs. It is clear that not only criteria but also bias restricts an objective evaluation. Although energy cost of compression has been noticed in some researches [10–13,16,18,20,23], a proper evaluation of data compression is still desired.

### 2.3. Researches on Compression Evaluation

In the literature which is most closely related to our own [26], several off-the-shelf lossless compression methods for mobile devices were reevaluated and tested on a StrongARM SA-110 processor. According to this work, the most popular coding algorithms were objectively compared under a uniform platform. Moreover, a more energy-efficient coding scheme was proposed based on the comparison results of energy costs. This work enlightened us on a fair and comprehensive evaluation of data compression; however, most of the algorithms in [26] were infeasible for sensor nodes.

Five different types of compression methods were summarized in [27], which were all designed for sensor nodes. The mentioned algorithms covered a wide range of characteristics, including lossy and lossless, one-dimensional and multi-dimensional, temporal and spatial. Whereas each method in [27] was focused on a given application, no comparison between them was reported owing to their different backgrounds.

Our work is aimed to establish a relatively objective environment for data compression evaluation in WSNs. Thus, compression algorithms, in special for sensor nodes, are selected; and the performance is assessed which is focused on the energy consumption. To our best knowledge, it is the first time data compression has been evaluated systematically and objectively from the point of view of energy efficiency in WSNs. The introduction of energy information in the evaluation represents the biggest difference between our work and the previous ones. It should be advisable to pay attention to our evaluation results before new designing algorithms or choosing existing ones.

## 3. Evaluation Principle and New Criterion

In this section, the selected compression algorithms are introduced briefly and the new evaluation criterion is proposed.

### 3.1. Background and Basic Concepts

Two basic concepts are mentioned in this paper: compression ratio and peak error. Compression ratio, denoted by *R_c_*, is defined as a ratio of two data volumes:

(1) $$R_{c}=\frac{\text{Volume}\;\text{of}\;\text{Compressed}\;\text{data}}{\text{Volume}\;\text{of}\;\text{Raw}\;\text{data}}$$

It is obvious that the smaller *R_c_*, the better compression effect. Peak error (*e_P_*) is one form of compression error, which is formulated as:

(2) $$e_{P}=\mathrm{Max}|y(n)-x(n)|$$

It indicates the maximum difference between raw data (*x(n)*) and the reconstructed one (*y(n)*), where *n* is sample number.

As mentioned in Section 2, there are several forms of compression error representation. Although RMS error and SNR seems more common in traditional compression methods, we think that peak error will be more appropriate for use in WSNs. Due to nodes’ limited computational capability, compression error seems inapplicable if it is defined as RMS or SNR. Besides the high complexity and large energy losses in error computation, compressed data need to be reconstructed at first, which will incur tremendous energy waste too. Since error requirement is generally given as an upper-bound beforehand by applications, more and more algorithms [8–14,17,18,21] use peak error owing to its simplicity and being able to avoid data reconstruction for verification of requirements. Thus, we consider peak error as the only error representation in this paper.

### 3.2. Compression Overview

We introduce off-the-shelf compression algorithms designed for sensor nodes in this subsection. Their characteristics are all threefold:

First, peak error is defined as the maximum data deviation accepted by each application. It is predetermined and informed to the sensor nodes *via* communication links.

Second, compression methods are tunable with respect to data accuracy. Changing *e_P_*, compression can be either lossless or lossy.

Third, algorithms belong to online compression with no training is needed.

(1) Predictive compression

In WSNs, environmental data show strong inter-relationships with each other in both temporal and spatial domains. Thus, various prediction models are established which predict current sample values in terms of the previous ones. An actual sample which is close to the predicted one will be removed from the raw data stream. Only the rest need to be transmitted. That becomes the basic principle of predictive compression.

Prediction based data compression was proposed well in [18], which covered almost all kinds of predictive compression suited for sensor nodes. To ensure the exhaustiveness, we choose them all in our evaluation. According to the diverse predictive models, the algorithms can be categorized into three groups, as shown in Table 2.

(2) Wavelet transformation

Wavelet transformation based on lifting scheme is popular used in WSNs, owing to its low complexity in implementation. A 5/3 wavelet presented in [10–13] was designed for compressing data in spatial domain; however, it also can be used in the temporal case conveniently. Originated from the Lazy wavelet, 5/3 wavelet introduces lifting scheme, an alternative method, to compute its coefficients. The whole process is divided into three steps: split, predict, and update. More details were provided in [11].

(3) Data fitting

By right of the continuity in variation, it is proper to replace a data stream with a form of line to decrease the total bits needed in representation. In WSNs applications, several algorithms are put forward based on this idea. We merge them into one group, and call it data fitting. Methods we select in this paper are LAA (Linear Approximation Algorithm) [17], PMC-MR (Poor Man’s Compression-Midrange), PMC-MEAN (Poor Man’s Compression—MEAN) [8], and LTC (Lightweight Temporal Compression) [9].

### 3.3. Evaluation Principle

To make an objective compression evaluation in WSNs, a proper criterion is needed, which focuses on the energy efficiency of each algorithm. We name it ESB (Energy-Saving Benefit) and denote it by *η*. ESB shows the energy savings introduced by compression algorithms. The expression is formulated as:

(3) $$\eta =\frac{E_{\mathrm{uncomp}}-E_{\mathrm{comp}}}{E_{\mathrm{uncomp}}}*100\%$$

According to the various topologies, we describe ESB with two levels: node level and network level. The biggest difference between them is the consideration of energy costs in data receiving. At the node level, ESB is formulated as:

(4) $$E_{\mathrm{uncomp}}=P_{\mathrm{TX}}(d)*L*T_{\mathrm{tran}}$$

(5) $$E_{\mathrm{comp}}=P_{\mathrm{MCU}}*L*T_{\mathrm{MCU}}\left(e_{P}\right)+P_{\mathrm{TX}}(d)*L*R_{c}\left(e_{P}\right)*T_{\mathrm{tran}}$$

So,

(6) $$\eta =\frac{P_{\mathrm{TX}}(d)*\left[1-R_{c}\left(e_{P}\right)\right]*T_{\mathrm{tran}}-P_{\mathrm{MCU}}*T_{\mathrm{MCU}}\left(e_{P}\right)}{P_{\mathrm{TX}}(d)*T_{\mathrm{tran}}}$$

At the network level, ESB is expressed as:

(7) $$E_{\mathrm{uncomp}}=\sum_{i=1}^{h}P_{\mathrm{TX}}\left(d_{i}\right)*L*T_{\mathrm{tran}}+\sum_{i=1}^{h-1}P_{\mathrm{RX}}*L*T_{\mathrm{tran}}$$

(8) $$E_{\mathrm{comp}}=P_{\mathrm{MCU}}*L*T_{\mathrm{MCU}}\left(e_{P}\right)+\sum_{i=1}^{h}P_{\mathrm{TX}}\left(d_{i}\right)*L*R_{c}\left(e_{P}\right)*T_{\mathrm{tran}}+\sum_{i=1}^{h-1}P_{\mathrm{RX}}*L*R_{c}\left(e_{P}\right)*T_{\mathrm{tran}}$$

So,

(9) $$\eta =1-R_{c}\left(e_{P}\right)-\frac{P_{\mathrm{MCU}}*T_{\mathrm{MCU}}\left(e_{P}\right)}{\sum_{i=1}^{h}P_{\mathrm{TX}}\left(d_{i}\right)*T_{\mathrm{tran}}+\sum_{i=1}^{h-1}P_{\mathrm{RX}}*T_{\mathrm{tran}}}$$

Meanings of the symbols mentioned are listed in Table 3. As shown in (3), *η* is related with the energy consumptions of two cases that one is transmitting the raw data directly, and the other is compressing data before transmitting. In the former case, almost all energy is spent on communication; while in the latter one, the total energy costs should include both computational and communication part.

In the communication part, *P_TX_* is intimately related to *d*. It is common that transmit power is configurable according to the distance. It is notable that, at the node level, we remove energy cost during data receiving from the communication part, which is reconsidered at the network level.

In the computational part, *P_MCU_* shows the power consumption when a microprocessor is in the active mode. *T_MCU_* and *R_c_* are highly dependent on the compression algorithm itself. Since the compression algorithms we selected are error-tunable, different values of *e_P_*, which are determined by applications, will affect both *T_MCU_* and *R_c_* directly and significantly.

From (6) and (9), we can see ESB includes the information of both compression ratio and time complexity explicitly. It is evident that neither compression ratio nor time complexity is competent for estimating compression algorithms fairly from the energy point of view.

In addition, compression error is also included by ESB. Its effect works on compression ratio and time overhead, which impacts *η* indirectly. To avoid unnecessary data transmission, data precision is usually pre-determined by each application. In other words, before sending compressed data to the sink, source nodes would know application demand in advance. In this case, compression error acts a role of adjudicator that evaluates whether requirement is satisfied.

Thus, the new evaluation criterion includes almost all the main metrics for evaluating compression, and reveals their internal relations by the way of energy evaluation. Besides, ESB additionally provides important information on whether data compression can bring energy savings or not. Just like our research presented in [28], compressions are not always energy efficient if the additional computational costs introduced by compression cannot be compensated by the communication energy savings. Ensuring energy-saving effect of compression is crucial in WSNs. Therefore, we add the energy costs in uncompressed case (*E_uncomp_*) to ESB.

## 4. Experimental Setup

### 4.1. Raw Data

WSNs have been universally used in environmental monitoring, including oceanography, atmospheric sciences, seismology, and so on. To guarantee an objective evaluation and remove bias in data selection, we choose actual and open datasets which are collected by sensor nodes and cover almost all common types and characteristics of environmental data. The datasets used in the test are summarized in Table 4.

### 4.2. Test Platform

We choose a MicaZ node as the test platform for compression evaluation. It is commonly used in WSNs. The processor is an 8-bit Atmel ATmega128L microcontroller. To be fairer, processor speed is fixed at 8 MHz. As the results shown in [33], supply current of processor is nearly constant in active mode. So, we consider *P_MCU_* as a fixed value in the evaluation. In MicaZ node, a CC2420 unit is responsible for communicating with other nodes. It is a single-chip RF transceiver that operates at 2.4 GHz. According to [34], the data transmission rate of a MicaZ node is up to 250 kbps. Besides, transmit power is configurable; in that case, CC2420 can be powered down by setting control register when communication distance is short. In the evaluation, we assume that source nodes send information in the range from 60 to 100 m, and the transmit power level is set to 31.

### 4.3. Methodology and Relevant Assumption

At the node level, network topology is assumed as a simple single-hop network. Source nodes send data to a powerful sink directly. In that case, energy costs in data receiving are no need to be considered. At the network level, it is a multi-hop network. Compression affects the energy consumptions in both transmission and reception. All compression algorithms are reimplemented and recompiled for the execution bias avoiding. *T_MCU_* is obtained by ATMEL AVR Studio [35]. Evaluation parameters mentioned are listed in Table 5.

## 5. Evaluation Results

To demonstrate the difference between the new criterion and the traditional ones, we show the evaluation results of all of them. For clearness, we summarize compression algorithms in Table 6. They are classified into three groups with different parameters. *N* denotes the number of historical data used for prediction modeling; smoothing coefficient *α* is selected based on the trends in data.

### 5.1. Compression Ratio

(1) Preferences in predictive compression

**Figure 1.**
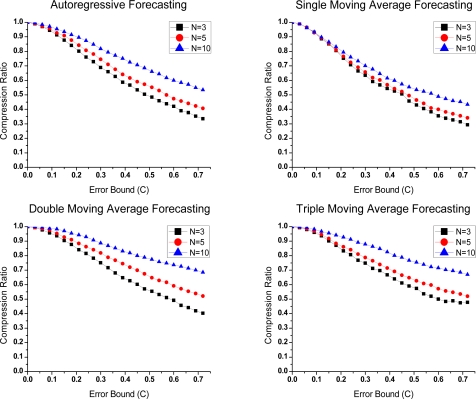
Compression ratio-Error bound curves of Group 1 under different parameters *N.*

**Figure 2.**
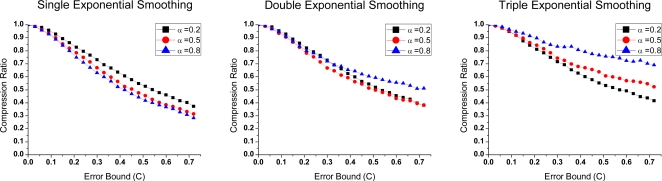
Compression ratio-Error bound curves of Group 2 with different parameters *α*.

In Groups 1 and 2, *N* and *α* are set to three different values, respectively. For the sake of conciseness, we show the test results under ambient temperature in Figures 1 and 2. Similar results can be obtained with the other datasets. In the figures, error bound (*e_P_*) describes application requirements of data precision. With its increase, all algorithms achieve lower compression ratioa owing to the improvement in forecast accuracy.

In Figure 1, a better compression effect is obtained when *N* is equal to 3. This can be attributed to the data characteristics and its short training period. As mentioned in [18], models need to be established before predicting. Parameter *N* determines the accumulated number of data for modeling. By right of the strong correlation in data, only a few historical samples are needed for a successful prediction. Since the amount of raw data is identical in the three conditions, the larger *N* is set, the more compressed data is left, which evidently worsens the compression effect.

In addition, compression ratio differences will be enlarged as the error bound increases. In large error bounds, more data can be eliminated from the raw data stream. *N* has more effects on compression ratio.

In Figure 2, the optimal *α* are different in the methods. In single exponential smoothing, the compression ratio is slightly lower if *α* is 0.8. The smoothing coefficient *α* reflects the influence degree of previous data in a prediction. Larger *α* indicates strong correlation in the data. Thus, a higher forecast accuracy is obtained.

The biggest difference between single exponential smoothing and the other two is that trend variation cannot be shown in the single one. As a result, in the other two methods, higher forecast accuracy is obtained due to the additional information. Meanwhile, *α* is decreased with the contribution brought by this improvement.

(2) Compression ratio comparison

**Figure 3.**
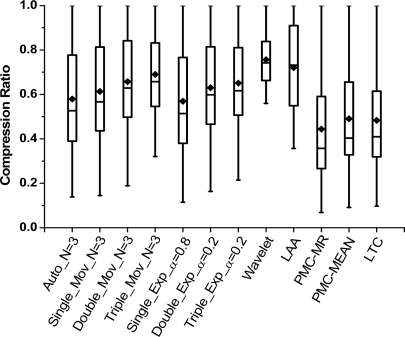
Compression algorithms comparison in compression ratio.

Compression ratios (*R_c_*) of all algorithms are shown in Figure 3. We test them under different data types and error bounds. The statistic information is developed by using quartile analyses. The mean is marked as a solid diamond. Each algorithm in Groups 1 and 2 is presented in the best case of the three.

In the figure, PMC-MR obtains the best compression effect of them all, while wavelet transformation is slightly worse than the others. In Group 1, autoregressive forecasting is better than the other three; in Group 2, single exponential smoothing is the best. It means simple model is competent for the test data. Wavelet transformation we use is one-level 5/3 wavelet. In this case, only half of the data (namely high frequency part) is compressed, which evidently limits its compression effect.

### 5.2. Compression Complexity

(1) Preferences in predictive compression

**Figure 4.**
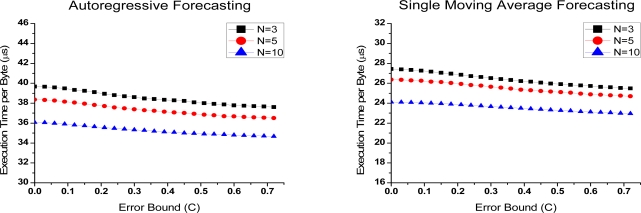

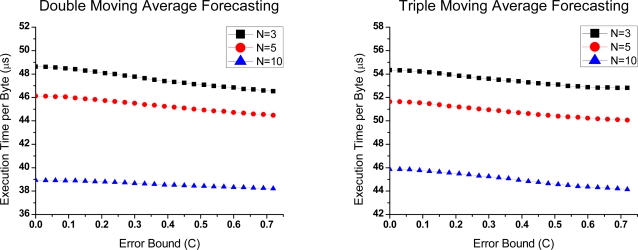
Execution time per byte-Error bound curves of Group 1 under different parameter *N.*

**Figure 5.**
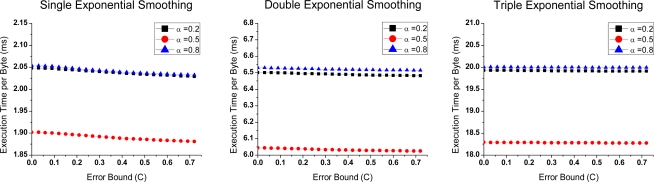
Execution time per byte-Error bound curves of Group 2 under different parameter *α*.

Figures 4 and 5 show the time overheads on compressing per byte (*T_MCU_*) of Groups 1 and 2. It is derived from the total time spent on compressing.

In the figures, *T_MCU_* has a similar trend as *R_c_* when the error bound increases. In the predictive compression, real sample should be added into a transmit queue once the deviation between the real and predicted one is larger than error bound. More operations are needed before transmitting. Thus, a less compressed data means a smaller *T_MCU_*.

In Figure 4, time overhead is lower if *N* is larger. In the small *N*, more data need to be predicted. The operation time correspondingly increases. In Figure 5, the lowest costs is obtained when *α* is equal to 0.5. In this case, division is replaced with shift operation, which requires less time consumption.

In Figure 5, *T_MCU_* is on the order of milliseconds. That is far longer than Group 1. It shows that algorithms consume a lot of time on division operations. As mentioned above, transmission of one byte needs 32 μs; however, the algorithms in Group 2 need several milliseconds of compression per byte. It is no doubt that compression is superfluous in this situation, because no energy savings will be obtained in any *α*.

(2) Compression complexity comparison

**Figure 6.**
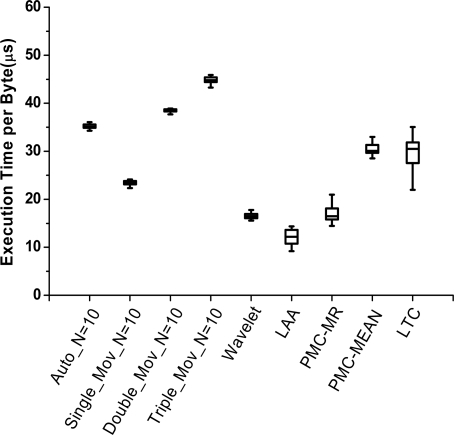
Compression algorithms comparison in execution time per byte.

Because of the high time overheads in Group 2, we eliminate them from the time comparison. In Figure 6, LAA has the shortest time overhead due to its low computational complexity with no division. Similar results are obtained in wavelet and PMC-MR, where shift operation is used instead of division.

### 5.3. ESB of Compression

(1) Preferences in predictive compression

**Figure 7.**
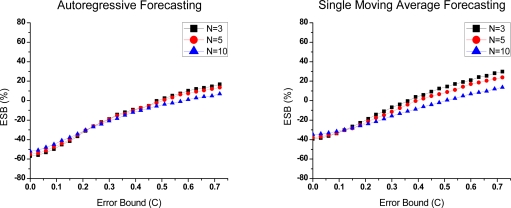

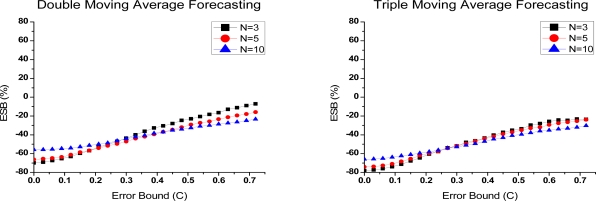
ESB-Error bound curves of Group 1 under different parameter *N* at the node level.

**Figure 8.**
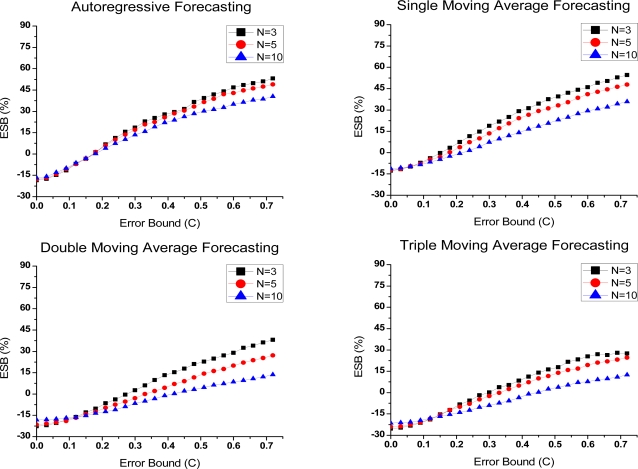
ESB-Error bound curves of Group 1 under different parameter *N* at the network level.

Due to the high time overhead in Group 2, it is hard to save energy by compression in common cases. Thus, we eliminate them from the ESB evaluation. ESB at the node and network level in Group 1 is presented in Figures 7 and 8. At the network level, the hop count (*h*) is 2. As shown in the figures, with the improvement of both compression ratio and execution time, ESB rise sharply when error bound increases. Either at the node level or network level, ESB is a little bit better when *N* is equal to 3. Although compression ratio is obviously superior in this case, the advantage is weakened by the drawback in the time overhead. Especially at the node level, the computational energy cost has a great impact on ESB. Moreover, it is noteworthy that compression saves the total energy only in the large error bounds.

(2) ESB comparison

**Figure 9.**
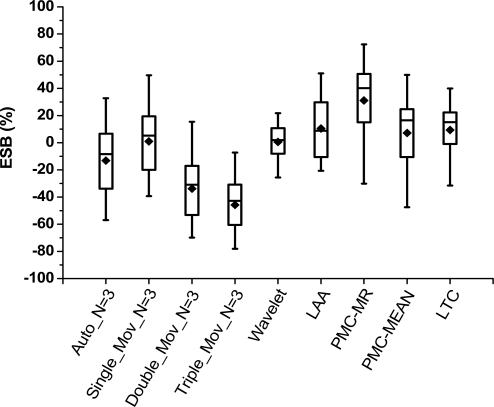
Compression algorithms comparison in ESB at the node level.

**Figure 10.**
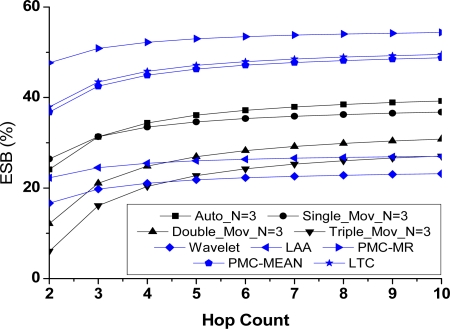
Compression algorithms comparison in ESB at the network level.

Except the three exponential smoothing forecasting ones, the remaining algorithms are evaluation based on ESB at the node and network level. The results are shown in Figures 9 and 10. The parameter N of Group 1 is set to 3. At the node level, the comparison result is shown using quartile analyses; at the network level, the average values of ESB under the different hop counts (*h*) are recorded.

It is clear that we obtain new comparison results which are different from the compression ratio and time complexity. Mainly owing to the excellent compression ratio and relatively low computational complexity, PMC-MR achieves the best energy-saving benefit among all algorithms listed. At the node level, it provides an average energy savings of 30% and the highest savings is as high as 70%. The probability that PMC-MR saves the total energy is higher than 75%. At the network level, ESB raises to 50% with the increase of hop counts.

It is worth mentioning that ESB of LAA is second only to PMC-MR at the node level. According to Figure 3, the compression ratio of LAA is not as good as that of the other algorithms. However, owing to its short execution time, LAA obtains a higher energy-saving benefit even than the algorithms with a lower compression ratio. It indicates that, viewed from the energy efficiency of compression, a low computational complexity could make up for the lack of the compression ratio. Nevertheless, LAA loses its advantage at the network level, as shown in Figure 10. In that case, the compression ratio has more effects on energy costs and more energy savings in communication benefit from it. With the increase of hop counts, the proportion of communication energy consumptions becomes large, while the influence of computational complexity on energy savings is smaller.

On the other hand, the algorithms show possibilities of introducing additional energy consumptions, especially at the node level. It mainly appears in the small error bounds, because at those moments, compressing data cannot save enough energy to offset the additional costs in computation, which makes compression unnecessary.

## 6. Adaptive Compression Arbitration System

As shown in Section 5, ESB is not always positive. In other words, data compression in WSNs is not always beneficial to energy conservation due to the additional computational energy dissipations. Thus, a low overhead method is needed as an assistant mechanism to avoid unnecessary losses in compression.

### 6.1. System Description

**Figure 11.**
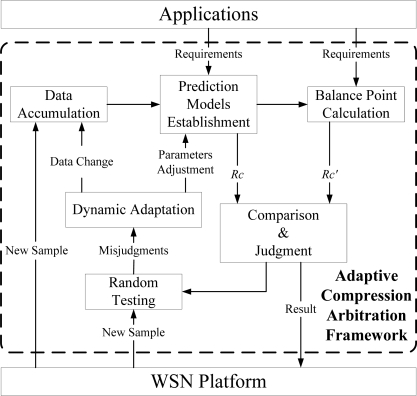
Framework for adaptive compression arbitration system.

An adaptive compression arbitration system is proposed with its framework shown in Figure 11. This system predicts the probable energy savings of compression to make a decision on whether to compress data before transmitting. The whole procedure is divided into three steps:

1. Prediction modeling
  Before the arbitration, two models are established on-line to predict the compression ratio and the compression time. Information about the compression ratio and execution time for various datasets and application requirements is recorded for each prediction model. Since it is an on-line modeling, only a few samples are used allowing for saving energy.
2. Compression evaluation
  After the modeling, the compression arbitration calculates a probable compression ratio for the given accuracy requirement and the corresponding time overhead based on the models. Then, the balance point between loss and benefit is estimated in the form of a compression ratio. Comparing the two kinds of compression ratio, the system draws a conclusion about whether compression will produce energy savings or not in the “comparison and judgment” sub-module. The feedback result is subsequentially applied to control the behavior of data processing (compression before transmission or direct transmission).
3. Adaptive modification
  In this step, several samples are randomly selected for the verification of judgment accuracy. Once the target sample is given, its actual compression ratio and time overhead are measured for evaluating whether data compression is beneficial for energy savings. If the evaluation result is different from that of arbitration system, parameter modification is realized *via* remodeling with the new data accumulated.

### 6.2. Experimental Results

The adaptive compression arbitration system is evaluated in a single-hop network with LTC as the test algorithm. Since the ultimate purpose of the arbitration system is reducing the total energy costs, we test the final energy savings provided by the new system under the different error bound levels and RF power levels. To show the efficiency of the system, two reference objects are used, which are the total energy costs for directly transmitting the raw data and the costs of compressing the data all along and then transmitting.

**Figure 12.**
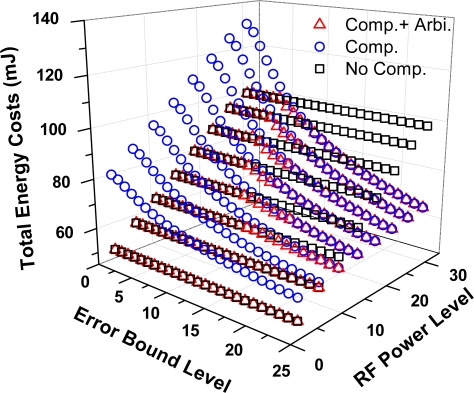
Energy efficiency of the adaptive compression arbitration system.

Energy consumptions for all three cases are presented in Figure 12. It is obvious that combining the new arbitration system with data compression, considerable energy savings can be obtained in most cases. The greatest saving is 33.4% of the cost of transmitting the data directly, which happens when both the error bound and the RF power are set to their maximums. In that case, the lowest compression ratio is achieved and the corresponding energy saving in communication has a significant influence on the total energy savings. Similarly, comparing to the case of always compressing the data, the highest percent savings is up to 39.2% when both the error bound and the RF power are set to their minimums. It is clear that, in that case, compression is no longer energy efficient, because it cannot save enough communication energy, while the additional cost in computation leads an unexpected energy waste.

## 7. Conclusions and Design Considerations

In the paper, many of the current tunable compression algorithms designed for WSNs are reevaluated based on the a criterion. Since all algorithms are aimed to be used in WSNs, which consider energy consumption as the first design element, the new criterion ESB reveals the performances of algorithms more objectively.

Although several indices proposed before are do well in the traditional compression evaluation, they are probably unable to be felicitously applied to WSNs. According to the comparison results, compression ratio and time complexity cannot express well the energy performance of the compression algorithms. Compression ratio only indicates the reduction in the data amount, which is numerically expressed in communication savings; time complexity only affects the additional computational energy consumptions for compression. That is to say, neither of these two indices can reveal the complete energy information about compression.

Besides the impartiality in algorithm evaluation, ESB can also be used to detect the case when compression wastes energy. It will probably happen if increased computational energy cannot be compensated by the decreased communication energy consumption. This information is much more important in both design and application. However, it seems hard to obtain from the other criteria.

Therefore, several design considerations are discussed based on the evaluation results:

First, computational energy brought by data compression is not always negligible. It may occur that compression costs much more energy, even if it has a satisfactory compression ratio. So, compression algorithm with a lower compression ratio does not mean it is the proper one for WSNs.

Second, different types of instructions have greatly different effects on the performance of algorithm. Especially in the division instruction, more execution time is needed, which deteriorates the energy efficiency of compression rapidly. It is obviously shown in exponential smoothing forecasting. So, the division instruction should be avoided in sensor nodes. We suggest using shift operation instead of it as much as possible.

Last but not least, an adaptive compression arbitration system is proposed with the enlightenment provided by the evaluation results. The system enhances the performance of compression algorithms by avoiding unnecessary energy losses. With this arbitration system, the greatest energy savings are 33.4% when directly transmitting the data and 39.2% when compressing all the data.

## Figures and Tables

**Table 1.** Summary of compression algorithms.

**Algorithm**	**Characteristic**	**Evaluation Index**	**Experimental Data**	**Platform**
DISCUS [5,6]	lossless spatial domain	distortion probability of decoding error	data created by a Gaussian model	N/A
PINCO [7]	lossless time domain	latency transmission cost	temperature map created	NS-2
PMC-MR PMC-MEAN [8]	lossy time domain	compression ratio	random walk data generated by a model	N/A
			sea surface temperature, salinity, shortwave radiation from TAO	
LTC [9]	lossy time domain	bytes savings memory usage	environmental data from Continuous Monitoring System	Mote node
Distributed Wavelet [10–13]	lossy spatial domain	SNR, bit rate, energy cost (ignore computational part)	data created by a second order AR model [10–13]	StrongARM
			temperature data on the Great Duck Island [13]	SA-1100
RACE [14]	lossy time domain	compression ratio compression error	environmental data from TAO	N/A
DPCM [15]	lossless time & spatial domain	coding gains	autoregressive source, acoustic source, weather data from NCDC	N/A
S-LZW [16]	lossless time domain	compression ratio execution time energy consumption	real data from SensorScope, Great Duck Island, ZebraNet, Calgary Corpus Geo	Tmote Sky ZebraNet node
LAA [17]	lossy time domain	compression ratio mean square error	real temperature data from Australian Bureau of Meteorology	N/A
Forecast-based Compression [18]	lossy time domain	successful rate of prediction energy cost	real temperature data	PowerTO SSIM
bzip2, zlib, LZW, Wavelet, ADPCM [19]	lossless time domain	compression ratio block size	acceleration data from MTx sensor	laptop
Gzip [20]	lossless time domain	compression ratio, energy cost execution time	six software modules	Tmote Sky
Top-down piecewise linear approximation [21]	lossy time domain	compression ratio time complexity	time series data collected from one sensor node	C++
LZO, bzip2, Gzip, rar, rzip [22]	lossless time domain	buffer size compression ratio time delay	trace-file from University of Crete	Intel Core2 Duo
Swinging Door [23]	lossy time domain	compression ratio mean square error energy cost (communication part)	real data from gas injection monitoring	NS-2 CC2520
COPE & DISCUS [24]	lossless spatial domain	compression ratio	N/A	N/A
2D-DCT [25]	lossless time & spatial domain	amount of data transmission average error	indoor temperatures collected by sensor nodes	MicaZ Mote

**Table 2.** Types of predictive compression.

**Type**	**Predictive Model**	**Predictive Algorithm**
Autoregression	AR Model	Autoregressive Forecasting
Moving Average	MA Model	Single Moving Average Forecasting
		Double Moving Average Forecasting
		Triple Moving Average Forecasting
Exponential Smoothing	ARMA Model	Single Exponential Smoothing Forecasting
		Double Exponential Smoothing Forecasting
		Triple Exponential Smoothing Forecasting

**Table 3.** List of symbol representation.

**Symbol**	**Signification**
*E_uncomp_*	Total energy costs without compression
*E_comp_*	Total energy costs with compression
*e_P_*	Error tolerance
*d*	Communication distance
*L*	Volume of raw data
*h*	Hop count
*R_c_*	Compression ratio
*T_tran_*	Time overhead on transmitting one byte
*T_MCU_*	Time overhead on compressing one byte
*P_TX_*	Transmit power
*P_RX_*	Receive power
*P_MCU_*	Computation power

**Table 4.** Types of datasets.

**Type**	**Notation**	**Application Background**	**Unit**
Air Temperature	AT		C
Sea Level Pressure	SLP	Tropical Atmosphere Ocean Project [29]	hPa
Relative Humidity	RH		%
Spectral Acceleration	PSA	The Pacific Northwest Seismograph Network [30]	pctg
Gage Height	GH	NWIS web water data [31]	feet
Farm Temperature	FT	The Georgia Automated Environmental Monitoring Network [32]	°F

**Table 5.** Values of parameters.

**Symbol**	**Value**	**Remark**
*d*	60–100 m	N/A
*P_TX_*	57.42 mW	PA_Level=31
*P_RX_*	62.04 mW	N/A
*T_tran_*	32 μs	250 kbps data rate
*P_MCU_*	26.4 mW	8 mA current draw

**Table 6.** Summary of algorithms evaluated.

**Group**	**Algorithms**	**Parameter**
Group 1	Autoregressive Forecasting	*N*
	Single Moving Average Forecasting	
	Double Moving Average Forecasting	
	Triple Moving Average Forecasting	
Group 2	Single Exponential Smoothing Forecasting	*α*
	Double Exponential Smoothing Forecasting	
	Triple Exponential Smoothing Forecasting	
Group 3	5/3 Wavelet	N/A
	LAA	
	PMC-MR	
	PMC-MEAN	
	LTC	
